# Identification of Novel Vascular Genes Downstream of Islet2 and Nr2f1b Transcription Factors

**DOI:** 10.3390/biomedicines10061261

**Published:** 2022-05-27

**Authors:** Ru-Fang Li, Yi-Shan Wang, Fu-I Lu, Yi-Shan Huang, Chien-Chih Chiu, Ming-Hong Tai, Chang-Yi Wu

**Affiliations:** 1 Department of Biological Sciences, National Sun Yat-sen University, Kaohsiung 804, Taiwan; fool74895@gmail.com (R.-F.L.); ki200412@yahoo.com.tw (Y.-S.W.); a0928020114@gmail.com (Y.-S.H.); cchiu@kmu.edu.tw (C.-C.C.); mhtai@faculty.nsysu.edu.tw (M.-H.T.); 2 Doctoral Degree Program in Marine Biotechnology, National Sun Yat-sen University, Kaohsiung 804, Taiwan; 3 Doctoral Degree Program in Marine Biotechnology, Academia Sinica, Taipei 115, Taiwan; 4 Department of Biotechnology and Bioindustry Sciences, National Cheng Kung University, Tainan 701, Taiwan; fuilu@mail.ncku.edu.tw; 5 The iEGG and Animal Biotechnology Center, National Chung Hsing University, Taichung 402, Taiwan; 6 Department of Biotechnology, Kaohsiung Medical University, Kaohsiung 807, Taiwan; 7 Institute of Biomedical Sciences, National Sun Yat-sen University, Kaohsiung 804, Taiwan; 8 Institute of Medical Science and Technology, National Sun Yat-sen University, Kaohsiung 804, Taiwan

**Keywords:** islet2, nr2f1b, transcriptome, follistatin a (*fsta*), vascular development, zebrafish

## Abstract

The genetic regulation of vascular development is not elucidated completely. We previously characterized the transcription factors Islet2 (Isl2) and Nr2f1b as being critical for vascular growth. In this study, we further performed combinatorial microarrays to identify genes that are potentially regulated by these factors. We verified the changed expression of several targets in *isl2/nr2f1b* morphants. Those genes expressed in vessels during embryogenesis suggested their functions in vascular development. We selectively assayed a potential target *follistatin a (fsta*). Follistatin is known to inhibit BMP, and BMP signaling has been shown to be important for angiogenesis. However, the *fsta*’s role in vascular development has not been well studied. Here, we showed the vascular defects in ISV growth and CVP patterning while overexpressing *fsta* in the embryo, which mimics the phenotype of *isl2/nr2f1b* morphants. The vascular abnormalities are likely caused by defects in migration and proliferation. We further observed the altered expression of vessel markers consistent with the vascular defects in *(fli:fsta)* embryos. We showed that the knockdown of *fsta* can rescue the vascular defects in *(fli:fsta)* fish, suggesting the functional specificity of *fsta*. Moreover, the decreased expression of *fsta* rescues abnormal vessel growth in *isl2* and *nr2f1b* morphants, indicating that *fsta* functions downstream of *isl2*/*nr2f1b*. Lastly, we showed that Isl2/Nr2f1b control vascular development, via Fsta–BMP signaling in part. Collectively, our microarray data identify many interesting genes regulated by *isl2/nr2f1b*, which likely function in the vasculature. Our research provides useful information on the genetic control of vascular development.

## 1. Introduction

The proper functioning of the cardiovascular system depends on the precise formation of blood vessels that were established in an evolutionary conserved manner in vertebrates. Vascular endothelial cells are differentiated from angioblasts that originated in the lateral plate mesoderm. The main axial vessels formed by the process of vasculogenesis, which involves the specification and differentiation of angioblasts, as well as their migration and aggregation into vascular cords [1,2]. Then, angiogenesis involves sprouting, migration, and proliferation of differentiated vascular endothelial cells, which form a vessel plexus [3,4,5]. The zebrafish model has been effective in identifying the molecules and signals responsible for blood vessel growth and patterning during development [6,7,8,9,10].

Recent studies have identified genes and singal pathways to regulate artery and vein specification of vasculogenesis in various vertebrates [11,12]. In zebrafish embryos, arterial specification is mainly controlled by vascular endothelial growth factor (Vegf) activating Notch signals via PLC-r-PKC-MEK pathways [13,14,15,16]. Venous cells expressing different markers indicated a distinct genetic regulation via PI3K/AKT signaling in angioblasts to enhance vein specification [17,18]. However, the precise mechanism of signaling interactions during vasculogenesis is not fully understood. After the artery and vein have formed, VEGF–Notch signaling continuously plays a critical role in ISV growth and patterning, including the specification of tip cells and stalk cells, as well as the proliferation and migration of endothelial cells [19,20,21]. Meanwhile at the tail region, a distinct mechanism of angiogenesis from ISV growth, BMP signaling, promotes endothelial progenitor cells sprouting from the posterior cardinal vein (PCV), as well as their proliferation and migration to form a CVP network [22]. Therefore, a complex coordination among molecules and signals has a critical role in regulating endothelial cell fates and behaviors during angiogenesis. However, the molecules that are required for ISV and CVP formation have yet to be fully documented. Identification of the novel regulators during the developmental processes will help in the understanding of vascular patterning and could serve as new therapeutic targets.

Transcription factors play a central role in the regulation of vascular development after receiving signals [23,24]; however, little is known about vein identity and ISV patterning in zebrafish. CoupTFII (Nr2f2) in mice, which plays a key role in venous identity and angiogenesis, has been identified [18,25], but *nr2f2* in zebrafish only plays a minor role in vein differentiation, as observed in other groups as well as from our observations [26,27]. Further, we previously identified the transcription factors Islet2 and Nr2f1b, which are required for the specification of the vein and tip cell identity, mediated by Notch pathways in zebrafish [28,29]. We, thus, hypothesized that Isl2 and Nr2f1b signaling pathways are involved in vein specification, tip cells’ proliferation, and migration during angiogenesis. To reveal the molecular mechanisms for how Isl2/Nr2f1b control vascular development, we used a genome-wide microarray approach to identify *nr2f1b*- and *isl2*-regulated genes in the vasculature.

In the current study, we knocked down *nr2f1b* and/or *isl2* as tools to identify novel genes that are potentially involved in vasculogenesis and angiogenesis. We validated expression patterns and changes in many interesting genes used by wt and *isl2/nr2f1bMO*. The identification of novel genes is critical to further understanding mechanisms of vascular development, which are regulated by Isl2 and Nr2f1b. We selectively assayed the functional relevance of one potential target, *follistatin a (fsta*), from the array data. Follistatin is a glycosylated protein and binds and neutralizes the actions of many members of the transforming growth factor-β family of proteins, such as activin, myostatin, TGFβ, and BMPs [30]. The functions of follistatin are known for oocyte maturation, neural morphogenesis, and skeletal muscle development [31,32]. However, not much is known about whether it functions in vascular development, although it has been reported to be involved in angiogenesis during skeletal muscle healing after injury and disease [33]. BMP signaling has been reported in vascular differentiation and angiogenesis via SMAD or non-SMAD pathways [34,35]. The deregulation of BMP signals has been implicated in vascular diseases and tumor angiogenesis [36]. However, the molecular mechanisms for how BMP signals regulate endothelial cell angiogenesis is not fully understood. Recent efforts in zebrafish studies reveal *dab2*- and *β-catenin*-mediated BMP signals that control CVP patterning [37,38].

We hypothesized that Isl2/Nr2f1b regulate ISV growth and CVP formation, mediated by *Fsta*–BMP signals, since BMP signaling has been shown to be involved in venous angiogenesis in zebrafish. Our data support *fsta* functioning in the vasculature downstream of *isl2/nr2f1b*. We also revealed that the interaction of *isl2/nr2f1b* and *Fsta*-BMP signals works in order to control vascular development. Together, our results have identified many vascular genes acting downstream of the Isl2/Nr2f1b pathway. Those targets might play important functions in regulating the networks of vascular development.

## 2. Materials and Methods

### 2.1. Zebrafish Husbandry and Chemical Treatment

Zebrafish *(Danio rerio)* and embryos were raised and maintained in a 28.5 °C fish room based on the Zebrafish Book guidelines [39]. The regulations were approved from the National Sun Yat-Sen University Animal Care Committee (approval reference #10231). Wild-type fish and transgenic fish lines *Tg(flk:eGFP)*, *Tg(fli1a:negfp)^y7^*, *Tg(gata1:dsRed)^sd2^*, and *Tg(kdrl:mCherry)^ci5^* [13,40,41,42] were used in this study from the Taiwan Zebrafish Core Facility at Academia Sinica. Embryos were cultured in E3 medium and dechorionation by incubation was performed in 2 mg/mL pronase (Sigma, St. Louis, MO, USA). 0.003% 1-Phenyl-2-Thiourea (PTU) (Sigma), which was added into the medium at 6 h post fertilization (hpf) to inhibit pigmentation. To investigate signal pathways, the embryos were treated with 15 μM of the VEGF inhibitor SU5416 (Calbiochem, Nottingham, UK) and 75 μM of the Notch signal inhibitor DAPT (Sigma) at a working concentration in E3 fish water at 6 hpf. Concentrations of 10 μM Dorsomorphin (DM, Calbiochem) or 40 μM DMH1 (Calbiochem) were used to inhibit BMP signals at 20 hpf. The control embryos were cultured in 0.3% DMSO medium.

### 2.2. Morpholinos and Tol2 DNA Injection

Gene expression knockdowns were performed through an injection of morpholino antisense oligonucleotides (MOs) (GeneTools LLC, Corvallis, OR, USA). Morpholinos were injected into embryos around the 1–2 cell stage using a FemtoJet microinjector (Eppendorf, Germany). Morpholinos specifically targeted to *nr2f1b* and *islet2* were described in [28,29]. Translation-blocking morpholinos targeting the ATG start codon of *fsta* or targeting the exon3–intron3 boundary to interfere with the splicing of *fsta* (*fsta*^e3i3^ MO) (both MOs are gifts from Dr. Waskiewicz; [43]) were injected with 8 ng.

*fsta* e3i3MO sequence: 5′-TGTGTTACCTACTTTTGCATTTGCC-3′

*fsta* ATGMO sequence: 5′-CGCTTTAGCATCCTTAGCATGTTTA-3′

To generate *fli:fsta*-overexpressing embryos, we used the Tol2kit vectors with the multisite Gateway cloning system (Invitrogen) to make expression constructs. *Fsta* coding region franking with *attb1/b2* sequences was amplified using the following primers to make pDONR-*fsta*. *fsta*-f-attb1: 5′-GGGGACAAGTTTGTACAAAAAAGCAG-GCTCCACCATGCTAAGGATGCTAAAGCGTC-3′ and *fsta*-r-attb2: 5′-GGGGACCACTTTGTACAAGAAAGCTGGGTTTTACTTACAGTTGCAAGATCC-3′. A Gateway construct containing a 0.8-kb *fli1a* promoter fragment was described in [44]. About 100 pg of plasmid DNA and 25 pg of transposase mRNA were co-injected into 1-cell stage embryos. The GFP signals driven by cardiomyocyte light chain (CMLC) from the vector backbone allowed us to verify transient *(fli:fsta)* overexpressing embryos

### 2.3. Microarray Analysis

The total RNA of wildtype and *isl2* morphants or *nr2f1b* morphants at 24 hpf were extracted using an RNAeasy mini kit (QIAGEN, Valencia, CA, USA) labeled with Cy3 or Cy5 and hybridized to a Nimblegen zebrafish 385 k chip (Roche Applied Science) at the University of Idaho IBEST DNA Sequencing Analysis Core. To control for features and obtain the raw intensity data for each probe and each array, the NimbleScan software program (NimbleGen) was used to align a chip-specific grid. Chip images for each array were checked as to whether they had any significant spatial artifacts. Raw intensity data were analyzed into an R statistical computing environment for a quality check. In addition, the distributions of chip intensity and hierarchical clusters were compared as to whether any unusual global patterns existed. Each array then performed background correction and normalization. Lastly, we used the median polish procedure to summarize all the probes contained within each probe set into a single expression value for each gene [45,46,47]. Probe sets with low expression levels were removed from the final number of statistical tests. Differential expression was analyzed using a linear model and an empirical Bayesian adjustment to the variances [48]. To control for the expected false discovery rate (FDR) given multiple tests, the Benjamini and Hochberg (BH) method was used [49]. Together, probe sets were considered statistically differentially expressed if they had a BH adjusted *p*-value of < 0.05. The average ratios of expression values of *isl2* morphants vs. control or *nr2f1b* morphants vs. control were calculated from three experiments. We chose an arbitrary cut-off value of an expression fold change ≥2.5 or <0.5 on both arrays (Table 1 and Table 2). Results were listed by the fold change from highest to lowest.

### 2.4. RNA Extraction and cDNA Preparation

Total RNA was prepared from certain stages (15S, and 24, 30, or 48 hpf) of embryos using the RNeasy mini extraction kit (Qiagen) according to the manufacturer’s instructions. To generate cDNA, 1 μg of total RNA was used as template, using a mix of oligo-dT primer (Roche) and the RT-reverse transcriptase (Roche) according to the manufacturer’s instructions.

### 2.5. Real-Time RT-PCR Analysis

Real-time quantitative PCR was assayed using SYBR Green Master mix (Roche) and the LightCycler 96 real-time PCR system (Roche) and relative cDNA levels were measured and calculated using the LC96 software (Roche), and adjusted to the internal control β-actin levels based on the ΔΔC_t_ method. The experiment was performed as biological triplicates. Real-time PCR primers were designed and listed in the Supplemental Table S1.

### 2.6. Whole-Mount In Situ Hybridization and Frozen Sections

Whole-mount in situ hybridization followed the protocol based on Thisse et. al. [50]. In-situ probes were obtained by PCR amplifying about 0.5~1 kb using the primers described in the Supplemental Table S2, as well as in vitro transcription using T7 Polymerase (Roche) with a DIG-labeled RNA labeling kit (Roche). Briefly, embryos were fixed in 4% paraformaldehyde (PFA) for 16 h and switched to methanol at −20 °C. After rehydration, permeabilization with proteinase K, the probe was added to embryos in hybridization buffer at 65 °C overnight, then was washed and blocked with BSA. An AP-conjugated anti-Dig antibody was added and proceeded to react with NBT/BCIP substrate (Roche). After the reaction, embryos were embedded and photographed. For transverse sections, the embryos were fixed and embedded with Tissue-Tek O.C.T Compound (Sakura Finetek, USA), sectioned at 10 µm using a CM3050S cryostat (Leica) and photographed on an IX71 microscope (Olympus, Tokyo, Japan).

### 2.7. Image Acquisition and Processing

Embryos were mounted in 3% methyl cellulose (Sigma) or 1.5% low melt agarose (Invitrogen, Philadelphia, PA, USA), and images captured with a color digital, an AxioCam HRc camera, and AxioVision software (Carl Zeiss, Germany). Embryos for the confocal images were immobilized and embedded in 5% Tricaine in 1.5% low-melting-point agarose (Invitrogen) and images were collected on a Zeiss LSM700 confocal microscope, and processed in ImageJ software. Final figures were made using Adobe Photoshop.

### 2.8. Protein Extraction and Western Blotting

Zebrafish embryos were dechorionated and homogenized at 4 °C using RIPA lysis buffer (AllBio Science, Taiwan) with protease inhibitor cocktails (Roche) and phosSTOP (Roche). The lysates were collected, then sodium dodecyl sulfate (SDS) sample buffer was added, and boiled for 15 min. The proteins were separated by SDS-PAGE (polyacrylamide gel electrophoresis) and then transferred to a polyvinylidene fluoride (PVDF) membrane (GE Healthcare). The membranes were washed in TBS-T (Tris-buffered saline with Tween-20) and blocked with nonfat milk at room temperature for 1 hr. The membranes were then incubated with anti-SMAD1, anti-phospho-SMAD1, anti-Erk1/2, anti-phospho-Erk1/2 (Cell Signaling Technology, Boston, MA, USA), and anti-β-actin (Sigma) overnight at 4 °C. The membranes were washed with TBS-T and incubated with a horseradish peroxidase (HRP)-conjugated secondary antibody at room temperature for 2 h. The signal was developed by an ECL chemiluminescence (Bio-Rad, Hercules, CA, USA) reaction.

### 2.9. Statistical Analyses

Data are shown as the mean ± standard error (S.E.) or mean ± standard deviation (S.D.) for at least two independent experiments with biological triplicates. Statistical analyses were performed with GraphPad Prism software. The *p* values of group comparisons were obtained by using the unpaired two-tailed Student’s *t* test. Statistical significance was concluded when the *p* values were <0.05.

## 3. Results

### 3.1. Microarray Analysis of nr2f1b and isl2 Downstream Targets

**Figure 1:**
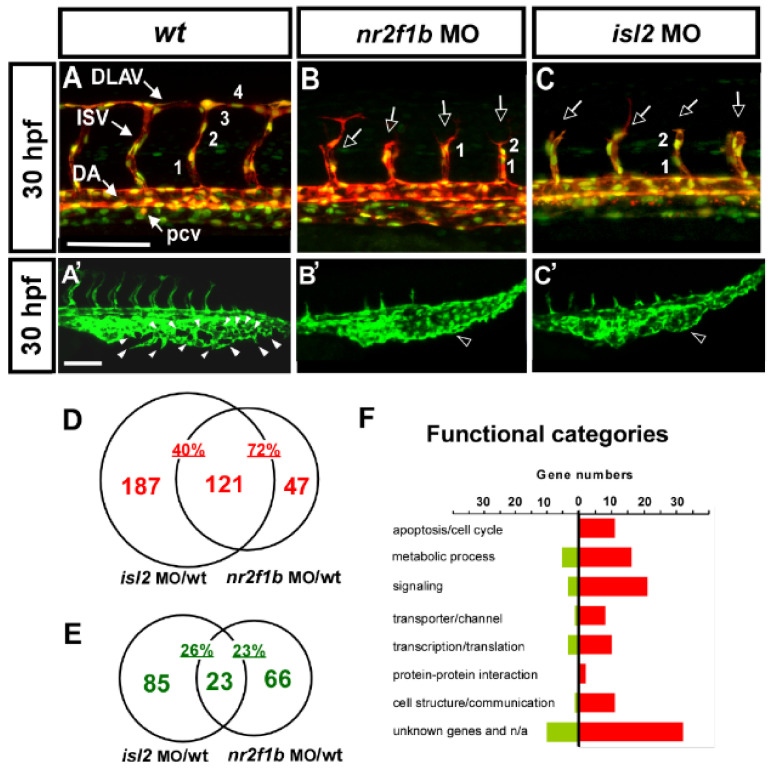
Loss of *nr2f1b* and *isl2* causes vascular defects, and transcriptome analysis identified genes regulated by Nr2f1b and/or Isl2. (**A**–**C**,**A′**–**C′**) knockdown of *isl2* or *nr2f1b* causes ISV growth stalling in the middle line of the somite, a reduction of the ISV endothelial cells, (**B**,**C**) and a mis-patterned vessel plexus at the CVP (**B′**,**C′**) at 30–32 hpf when compared to wt controls (**A**,**A′**). (**D**,**E**) Microarray data analysis in Venn diagrams show that there were 308 and 168 genes upregulated at *isl2* MO and *nr2f1b* MO, respectively, compared to wt, as well as 121 overlapping genes in both *isl2* MO and *nr2f1b* MO when compared to the wild type (**D**). (**E**) For the repression targets, 108 and 89 genes are downregulated at *isl2* MO and *nr2f1b* MO, respectively, compared to wt, and 23 genes are overlapped. (**F**) Functional analysis showed many genes involved in cell cycle regulation, signaling, transporter/channels, transcription, cell communication and protein–protein interactions. (ISV, intersegmental vessel; DLAV, dorsal longitudinal anastomotic vessel; DA, dorsal aorta; PCV, posterior cardinal vein; CVP, caudal vein plexus). Scale bars represent 100 μm.

Our previous showed the transcription factors Islet2 and Nr2f1b to be critical for the specification of the vein and tip cell, mediated by the Notch pathway in zebrafish [28,29]. The knockdown of *isl2* or *nr2f1b* impairs the vascular patterning of ISV and CVP (Figure 1A–C,A′–C′). At 30 hpf, in uninjected control embryos, ISV reached the DLAV at the dorsal region of the trunk (Figure 1A) and the CVP formed honeycomb-like structures via the sprouting and fusion of endothelial cells at the tail part (Figure 1A′). The loss of *nr2f1b* or *isl2* showed an ISV growth defect (Figure 1B,C *hollow arrows*) and a mis-patterning of the plexus at the CVP (Figure 1B′,C′ *hollow arrowhead*). In addition, the knockdown of *isl2* or *nr2f1b* resulted in reduced numbers of endothelial cells per ISV in the *Tg (kdrl:mCherry; fli1a:negfp ^y7^)* embryos, where GFP was expressed in the nucleus of endothelial cells and the mCherry, labeled on ISV cells (Figure 1A–C *numbers*). We hypothesize that Islet2 and Nr2f1b signaling pathways are involved in vein specification and tip cells’ proliferation and migration during angiogenesis. In this study, we used a genome-wide microarray approach to identify *nr2f1b*- and *isl2*-regulated genes in the vasculature, specifically in the patterning of ISV and CVP.

To assay transcriptome level changes after the loss of *nr2f1b* or *isl2*, we undertook microarray analysis and compared the gene expressions for *isl2* or *nr2f1b* morphants and wild-type embryos using 32,000 gene Nimblegen zebrafish chips (based on Zv8 genomic sequence assembly) and a full hybridization service from Nimblegen. The array was run in three biological replicates (i.e., independent morpholino injections). Data from the array were statistically analyzed and we chose an arbitrary cut-off value of an expression fold change of ≥ 2.5 or < 0.5 on both arrays (Table 1 and Table 2). The results were sorted by the fold change from highest to lowest. We found 79 potential targets to be up-regulated over 2.5-fold after the knockdown of *nr2f1b* and *isl2* transcription factors. On the other hand, Table 2 lists a downregulation of greater than 2-fold of the 23 potential targets controlled by *isl2* and *nr2f1b*.

We found a large portion of overlapping downstream targets for *nr2f1b* and *islet2*. There are 121 of 308 (40%) potential repression targets of isl2 and 121 of 168 (72%) potential repression targets of *nr2f1b* that are overlapped (Figure 1D). In addition, 23 of 108 (21%) and 23 of 89 (26%) are downregulated in *isl2* and *nr2f1b* morphants, respectively (Figure 1E). The high percentage of overlapping targets suggests that *nr2f1b* and *isl2* cooperatively regulate vascular development, likely in the same pathway. Functional analysis showed many genes involved in cell cycle regulation, signaling, transporter/channels, transcription, cell communication, and protein–protein interactions (Figure 1F). These functions are critical for the behavior of endothelial cells to form vessel networks [51,52].

**Table 1:** Genes that are up-regulated ≥2.5-fold in both *isl2* MO and *nr2f1b* MO.

Genbank/Array ID	Gene Symbol ^a^	Gene Name	isl2MO/wt ^b^	nr2f1bMO/wt ^b^
AI943081		voltage-dependent calcium channel	29.10	23.72
BC151913.1		receptor activity	14.18	19.74
BC066689.1	**cpn1**	carboxypeptidase N, polypeptide 1	12.40	10.85
ENSDART00000073432	RBL2	retinoblastoma-like protein 2	11.31	11.88
ZV700S00000110	casp8	caspase 8	10.15	10.17
ZV700S00000432	**lnx1**	ligand of numb-protein X1	9.90	9.92
ENSDART00000086206	ATXN1	ataxin-1/Spinocerebellar ataxia type 1 protein	9.23	12.45
ENSDART00000005808		calponin-1-like	8.49	9.00
ZV700S00000355	phlda3	pleckstrin homology-like domain, family A, member 3	8.25	8.03
ENSDART00000016263		probable G-protein-coupled receptor-like	7.50	3.21
OTTDART00000022443	**fsta**	follistatin a	7.41	6.36
TC254361	bbc3	bcl2 binding component 3	6.84	6.68
OTTDART00000030315	cdkn1a	cyclin-dependent kinase inhibitor 1A	6.57	5.01
OTTDART00000021597	**ftr82**	finTRIM family, member 82	6.50	7.25
ENSDART00000097728		similar to neurotoxin/C59	6.49	5.68
OTTDART00000027213	mdm2	murine double minute 2 homolog	6.48	8.07
OTTDART00000007800	**foxo3b**	forkhead box O3b Transcription factor	6.38	7.35
ZV700S00006205	RPS27L	ribosomal protein S27-like protein	5.74	4.68
OTTDART00000019467	tp53	tumor protein p53	5.74	5.44
BC072702.1	cyp3a65	cytochrome P450, family 3, subfamily A, polypeptide 65	5.61	5.60
OTTDART00000025224	**mmp2**	matrix metalloproteinase 2	5.22	5.99
ZV700S00003980	gadd45aa	growth arrest and DNA-damage-inducible, alpha, a	5.07	4.50
TC253576		Beta-1,3-glucosyltransferase-like	5.05	4.12
ENSDART00000034197	SUSD5	Sushi domain-containing protein 5	4.80	4.91
ZV700S00006317	igf2a	insulin-like growth factor 2a	4.68	4.46
OTTDART00000007033	fos	v-fos FBJ murine osteosarcoma viral oncogene homolog	4.54	4.82
BC056806.1	**gtpbp1l**	GTP-binding protein 1-like	4.30	4.23
BM571671		protein p8	4.25	5.95
ENSDART00000081980		LAG1 homolog, ceramide synthase 5-like	4.24	4.26
TC250557	slc4a2a	solute carrier family 4, anion exchanger, member 2a	4.23	2.93
OTTDART00000007990	**cx32.2**	connexin 32.2/cell junction	4.17	6.50
TC242899	crfb1	cytokine receptor family member b1	4.07	4.06
ENSDART00000097555	PAPPA	Pappalysin-1 Precursor	4.05	2.82
AW826461	sept8b	septin 8b Septin-like protein	3.98	3.29
ZV700S00005741	pik3r1	phosphoinositide-3-kinase regulatory subunit	3.98	3.61
TC245426	ect2	epithelial-cell-transforming sequence 2 oncogene	3.90	3.71
AI793818	chrngl	cholinergic receptor, nicotinic, gamma like	3.90	3.75
NM_131862	jag2	jagged 2	3.86	4.95
TC238338	otop1	otopetrin 1	3.85	3.23
OTTDART00000029706		similar to vertebrate doublecortin domain-containing 2, DCDC2	3.78	3.53
OTTDART00000025192	f2	coagulation factor II, thrombin/(prothrombin)	3.73	3.58
ZV700S00004623	cdkal1	CDK5 regulatory subunit-associated protein 1-like 1	3.72	2.89
OTTDART00000017384	aqp12	aquaporin 12	3.68	3.90
ZV700S00005526	sesn3	Sestrin 3	3.66	5.11
ZV700S00000935	hdac4	histone deacetylase 4 chromatin modification	3.65	3.01
OTTDART00000010432	ncl1	nicalin	3.52	3.28
ZV700S00003982	rlbp1b	retinaldehyde binding protein 1b	3.42	3.09
ZV700S00003517	rhoub	ras homolog gene family, member Ub	3.37	3.18
ZV700S00000580	ccng1	cyclin G1	3.36	3.40
BI984278_R; BI984278	taf1a	TATA box binding protein (TBP)-associated factor	3.32	2.70
OTTDART00000027172	cry-dash	cryptochrome DASH. (photolyase-like)	3.26	3.53
ZV700S00000920	phlda2	pleckstrin homology-like domain, family A, member 2	3.25	3.02
TC249636	C6	complement component 6	3.23	2.78
BQ419558		nardilysin isoform b (N-arginine dibasic convertase)	3.22	2.79
OTTDART00000030385	qdpra	quinoid dihydropteridine reductase	3.20	2.70
ENSDART00000049419		jagged2-like	3.18	3.93
ZV700S00004928	rasl11b	RAS-like, family 11, member B	3.16	3.33
ZV700S00003200	fabp1b.1	Fatty acid binding protein 1b	3.12	2.89
ENSDART00000059592	MEP1B	Meprin A beta	3.12	3.17
ZV700S00002841	**scarb2**	scavenger receptor class B, member 2	2.72	3.40
BC053278.1	**itm2bb**	integral membrane protein 2b	2.68	3.14
BI980126	SEC15L2	SEC15-like 1 isoform a	2.95	2.70
BC153975	rpz5	Rapunzel 5	2.90	4.01
BC064309.1	tmem38a	transmembrane protein 38A/cation channel transport activity	2.80	2.56
ENSDART00000065340	tlr9	Toll-like receptor 9/innate immune response	2.79	4.52
OTTDART00000017062		similar to vertebrate capicua homolog	2.78	3.11
NM_001007784	crygn1	crystallin, gamma N1	2.72	3.33
ENSDART00000080490		similar to peroxidasin	2.64	2.57
OTTDART00000025182	slc38a3	solute carrier family 38, member 3	2.54	2.71
NM_131156	krt5	keratin 5	2.54	2.68

^a^ Genes shown in bold were confirmed independently by in situ hybridization in Figure 2. ^b^ Expression rations are the average of three independent microarray experiments.

**Table 2:** Genes down-regulated ≥2-fold in both *isl2* MO and *nr2f1b* MO.

Genbank/Array ID	Gene Symbol ^a^	Gene Name	isl2MO/wt ^b^	nr2f1bMO/wt ^b^
ENSDART00000008778		extracellular-glutamate-gated ion channel activity	0.24	0.23
OTTDART00000007452	eef1db	translation elongation factor-1, delta,b	0.33	0.19
OTTDART00000028819	**nsdhl**	NAD(P) dependent steroid dehydrogenase-like	0.33	0.31
NM_213309	**stap2b**	signal transducing adaptor family member 2b	0.34	0.32
NM_001002580	pmepa1	prostate transmembrane protein, androgen-induced 1	0.36	0.37
ENSDART00000066927		N/A	0.37	0.41
BC085576	dhrs13a.1	oxidoreductase activity	0.37	0.39
NM_001082856	rorb	RAR-related orphan receptor B (NR1F2)	0.37	0.29
AL922441		N/A	0.37	0.38
CD605872_R		DNA helicase B	0.38	0.37
ENSDART00000027268	otpa	orthopedia homolog a	0.39	0.37
ENSDART00000074537	shisa9b	protein shisa-9B	0.39	0.30
ENSDART00000077671		similar to Aldehyde dehydrogenase 9 family, member A1 like 1	0.39	0.38
NM_001025174	fam110b	family with sequence similarity 110, member B	0.42	0.42
TC246456		metallocarboxypeptidase activity	0.42	0.29
ENSDART00000111589	hapln1a	hyaluronan and proteoglycan link protein 1a	0.43	0.45
ENSDART00000097296		similar to alpha-2,3-sialyltransferase ST3Gal I-r2	0.43	0.36
ENSDART00000058866	nes	Nestin	0.43	0.36
CR545462	**WASF1**	similar to vertebrate WAS protein family, member 1	0.44	0.34
ZV700S00001176	ccn1	cyclin I	0.45	0.47
NM_001003834	top2a	DNA topoisomerase (DNA) II alpha	0.45	0.35
BC164898.1	her4.1	hairy-related 4.1	0.46	0.42
NM_205585	**sfrp1a**	secreted frizzled-related protein 1a	0.46	0.43

^a^ Genes shown in bold were confirmed independently by in situ hybridization in Figure 2. ^b^ Expression rations are the average of three independent microarray experiments.

### 3.2. Verification of Expression Pattern of Several Potential Targets

**Figure 2:**
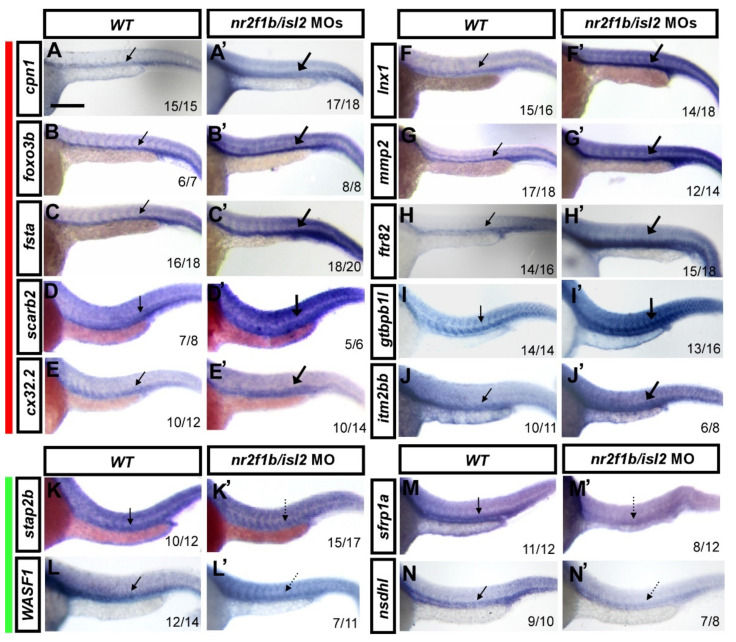
Verification of vascular-specific expression of potential targets in wt and double knockdown *isl2/nr2f1b* MOs by in situ hybridization. Embryos were injected with a combination of *isl2* and *nr2f1b* morpholinos (8 and 1.7 ng each) and gene expression was examined by in situ hybridization at 24 h post-fertilization. Representative wt control embryos are on the left of each panel and *isl2/nr2f1b* morpholino (MO)-injected embryos are on the right. ((**A**–**J**,**A′**–**J′**) red bar aside) The increased expression of (**A′**) *cpn1*, (**B′**) *foxo3b*, (**C′**) *fsta*, (**D′**) *scarb2*, (**E′**) *cx32.2*, (**F′**) *lnx1*, (**G′**) *mmp2*, (**H′**) *ftr82*, (**I′**) *gtpbp1l*, and (**J′**) *itm2bb* in the vasculature in *isl2/nr2f1b* morphants (thick arrows) compared to wild-type embryos (**A**–**J**). (**K**–**N**,**K′**–**N′** green bar aside) The reduced expression of (**K′**) *stap2b*, (**L′**) *WASF1*, (**M′**) *sfrp1a*, and (**N′**) *nsdhl* in the vessels in *isl2/nr2f1b* morphants (dash arrows) compared to wild-type embryos (**K**–**N**). These data consist of the transcriptome results. Values on the bottom indicate the number of embryos exhibiting a phenotype per the total number of embryos analyzed. Scale bar is 200 μm for all figures.

The morpholino knockdown of *isl2/nr2f1b* increased the expression of genes identified by *isl2* MO and *nr2f1b* MO transcriptome analysis. To independently validate expression changes of the most interesting genes, we verified whether the expression changes of the novel targets changed using whole-mount in situ hybridization at 24 hpf. We selectively analyzed ten up-regulated genes: *cpn1*, *foxo3b*, *fsta*, *lnx1*, *mmp2*, *ftr82*, *cx32.2*, *gtpbp1l*, *scarb2*, and *itm2bb*, and four down-regulated genes: *stap2b*, *WASF1*, *nsdhl*, and *sfrp1a*. Embryos were injected with a combination of *isl2* and *nr2f1b* morpholinos (8 and 1.7 ng, respectively) and gene expressions were examined by in situ hybridization at 24 h post-fertilization. An increased expression of *cpn1*, *foxo3b*, *fsta*, *scarb2*, *cx32.2*, *lnx1*, *mmp2*, *ftr82*, *gtpbp1l*, and *itm2bb* was observed in the vasculature in *isl2/* *nr2f1b* morphants (thick arrows) when compared to wild-type embryos (Figure 2A–J,A′–J′). A reduced expression of *stap2b*, *WASF1*, *sfrp1a*, and *nsdhl* in the vessels in *isl2/nr2f1b* morphants (dash arrows) was observed when compared to wild-type embryos (Figure 2K–N,K′–N′). This was consistent with the microarray results, which supports that the genes showed higher expression in uninjected wild-type embryos when compared to *nr2f1b*/*isl2* knockdown fish. The data also show the expression of genes in the vessels, suggesting their function in vascularization.

### 3.3. fsta Is Highly Conserved and Is Expressed in the Vasculature during Development

We selectively assayed the functional relevance of one potential target from the array data, *fsta*. Follistatin (Fst) is a known BMP antagonist related to axis formation during embryogenesis, and BMP signals have been shown to be important for angiogenesis, in order to form the CVP. However, no description of *fsta* functioning in vascular development is reported. We started to align follistatin amino acid sequences among vertebrates such as humans, mice, and zebrafish using ClustalW2 software. By comparing the amino acid sequences of zebrafish follistatin with orthologs from other species, a high percentage of conservation (>75%) of amino acids was shown (Supplementary Figure S1A,C). Zebrafish have a paralogous Fstb, which showed 79 and ~66% similarity to zFsta and mammalian FST, respectively. We also identified the putative TB ligand-binding domain (similarity = 95%), three F-like domains, and three Kazal-like domains (similarity = 98%), suggesting their role in protein–protein interaction (Supplementary Figure S1B). This information suggests the function of zebrafish Fsta has a similar function as mammalian FST. 

**Figure 3:**
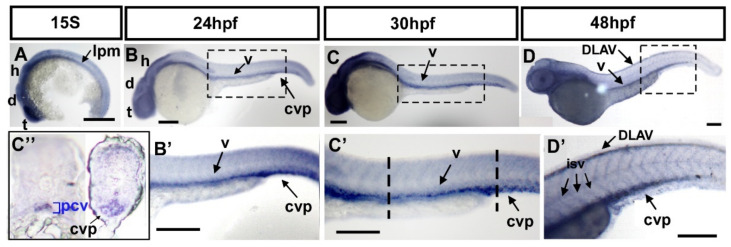
Expression pattern of *fsta* during zebrafish embryogenesis. (**A**) The expression of *fsta* is observed in the lateral plate mesoderm (lpm), the telencephalon (t), diencephalon (d), and hindbrain (h) at the 15S stage embryo. (**B**,**B′**) At 24 hpf, *fsta* is expressed in the head, as well as in vessels (v), and caudal vein plexus (cvp) of the trunk. (**B′**) is an enlargement of (**B**). (**C**,**C′**) At 30 hpf, *fsta* expression continues in the head, vessels (v), and CVP of the trunk. (**C′**) is an enlargement of (**C**). (**C″**) Cross sections of embryos from (**C**′) show that *fsta* is expressed in the posterior cardinal vein (pcv), and CVP. (**D**,**D**′) At 48 hpf, *fsta* expression in the head, vessels (v), DLAV, and CVP of the trunk. (**D′**) is an enlargement of (**D**). Scale bars in all figures represent 200 μm.

To investigate the function of *fsta* in vascular formation, we first determined the expression of *fsta* by in situ hybridization during zebrafish development. *fsta* is expressed in the lateral plate mesoderm (lpm), and the head area of the telencephalon (t), diencephalon (d), and hindbrain (h) at the 15 somite stage (S) (Figure 3A). Expression of *fsta* at the lpm is consistent with the place of the developing vessels. At 24–30 hpf, *fsta* is expressed in the head region, as well as the vessels (v) of the trunk and the CVP (Figure 3B,C,B′,C′). At 48 hpf, *fsta* expression can be detected in the head, vessels (v), ISV, DLAV, and CVP (Figure 3D,D′). Transverse sections of 30 hpf fish embryos show that *fsta* is expressed in the posterior cardinal vein (PCV) and CVP (Figure 3C″). The data show the expression pattern of *fsta* in blood vessels during zebrafish embryogenesis and indicate that *fsta* plays a role in vascular development.

### 3.4. Overexpression of the fsta Gene Causes Defects in Vascular Development

**Figure 4:**
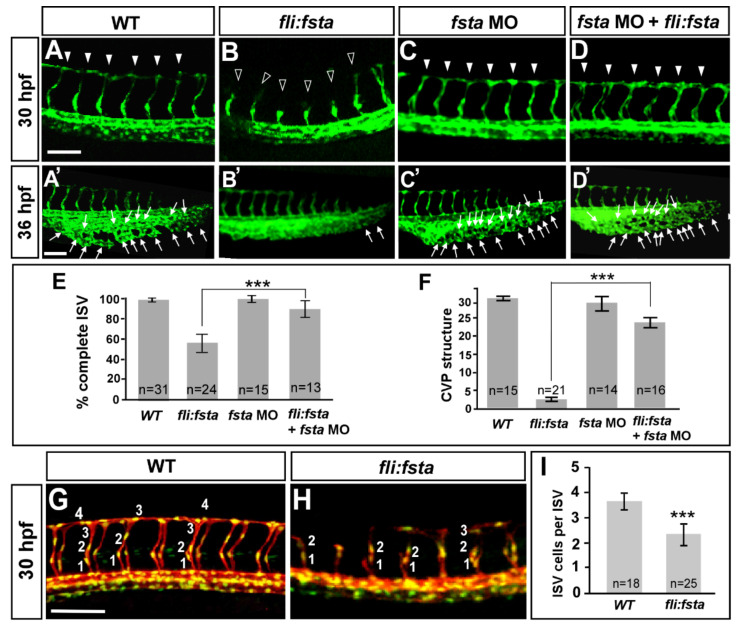
The overexpression of the *fsta* gene causes defects in vascular development. In uninjected control embryos, intersegmental vessels (ISV) reached the DLAV at the dorsal region from 24–30 hpf ((**A**), arrowheads) and the caudal vein plexus (CVP) formed honeycomb-like structures at the tail region from about 36–48 hpf ((**A′**), arrows). At the same stage, the overexpression of *fsta* driven by the fli promoter caused ISVs’ stalling at the mid-somite ((**B**), hollow arrowheads) and less/no honeycomb structure formation at the CVP (**B′**) Knockdown of *fsta* by morpholino had no obvious defect in the vasculature (**C**,**C′**), but rescued the defect of ISV stalling (solid arrowhead) (**D**) and restored the honeycomb structure at the CVP (**D′**)**.** (**E**) The quantification of the percentage of completed ISV at 30 hpf in wild-type embryos (97 ± 2, *n* = 31), (*fli:fsta)*-overexpressing embryos (54 ± 8, *n* = 24), *fsta* MO (97 ± 3, *n* = 15), and rescued embryos (84 ± 6, *n* = 13). (**F**) Quantification of loop formation (loops and sprouts) at the CVP at 36 hpf showed an 8-fold increase in the rescued embryos (23 ± 3, *n* = 16) when compared to (*fli:fsta*) embryos (3 ± 1, *n* = 21); *fsta* MO (28 ± 4, *n* = 14) has no difference when compared to wild-type embryos (30 ± 2, *n* = 15). (**G**,**H**) Imaging of endothelial nuclei in green and vessels in red at 30 hpf in wild-type controls and *(fli:fsta)*-overexpressing embryos using Tg(*kdrl:mCherry^ci5^*; *fli1a:nEGFP ^y7^)* double transgenic line. *(fli:fsta)* embryos showed reduced ISV nuclei numbers (**H**). (**I**) Quantification of ISV nuclei number in *(fli:fsta)* embryos (*n* = 25) when compared to wild-type controls (*n* = 18). Data are represented as means ± S.E. *** refers to *p*< 0.0001 by an unpaired Student’s *t*-test. Scale bars are 100 μm for (**A**–**D**,**A′**–**D′**,**G**,**H**).

We observed the increased expression of *fsta* during the knockdown of *isl2/nr2f1b*. Thus, we hypothesized that by overexpressing *fsta* in the embryos, we should see similar vascular defects as observed with the *isl2/nr2f1b* morphants. While overexpressing *fsta* by 200pg mRNA injection, we observed severe defects in axis formation and dorsalization (data not shown), which is consistent with the function of Fsta in dorsal–ventral formation [43]. Alternatively, we transiently overexpressed *fsta* in endothelial cells using the *fli1* promoter. In this way, we observed the similar vascular defects as *isl2* or *nr2f1b* morphants, including the uncompleted migration of ISV to the top of embryo (Figure 4B), the decreased number of ISV cells (Figure 4H), and reduced CVP loop formation via angiogenic sprouting and the fusion of endothelial cells (Figure 4B′). Specifically, at 30–36 hpf in the wt control, ISV reached the DLAV at the dorsal region of the embryo (Figure 4A) and the CVP formed loop structures at the tail (Figure 4A′). At the same stage, the overexpression of *fsta* driven by the fli promoter caused ~45% ISVs to stall at the mid-somite (Figure 4B, hollow arrowheads) and less/no honeycomb structure formation at CVP was observed (Figure 4B′). The knockdown of *fsta* by morpholino had no obvious defects on the vasculature (Figure 4C,C′), but rescued the defect of ISV stalling (solid arrowheads) about 30% of the time and restored the honeycomb structure ~8-fold at the CVP, which was significant (Figure 4D,D′). We also counted the numbers of endothelial cells per ISV in the *Tg (kdrl:mCherry^ci5^; fli1a:negfp ^y7^)* embryos and showed reduced ISV cells in (*fli:fsta*) fish (2.3 ± 0.4, *n* = 25 embryos) when compared to the wild-type control (3.7 ± 0.3, *n* = 18 embryos) (Figure 4G–I). Together, the overexpression of *fsta* phenocopying the loss of *nr2f1b* or *isl2* (Figure 1B,C,B′,C′) is consistent with our hypothesis that *fsta* functions in vascular patterning, and is negatively regulated by *nr2f1b* and/or *isl2*.

The overexpression of *fsta* resulting in vascular growth defects suggests the impairment of endothelial cellular migration, proliferation, or an increase in cell death. Our data showed a reduced completion of the ISV structure and ISV cells, suggesting that *fsta* negatively regulates ISV cell growth to contribute to vascular development, likely by regulation of the proliferation or migration of the cells. In addition, we also examined if there is more cell death in (*fli:fsta*) embryos. We showed that vascular defects in the (*fli:fsta*) embryo were not the result from non-specific cell death, as there was no significant increase in apoptosis in the vessels with overexpressed *fsta* when compared to wild type embryos, as observed by Acridine orange staining and a TUNEL assay (Supplementary Figure S2). 

Since edema and the blocking of circulation are common secondary effects of blood vessel impairment, we examined if these side effects occurred in the overexpressed *fsta* embryo. At 48–72 hpf, we found that the overexpression of *fsta* causes an increasing pericardial edema or circulation defects (Supplementary Figure S3A–F). The overexpression of *fsta* in transgenic *Tg (gata1:dsRed)^sd2^* embryos with dsRed-labeled blood cells allowed us to observe the circulation. The overexpression of *fsta* embryos showed 60% circulation defects with slower blood flow in the arteries/veins and/or less-to-no circulation at the ISV/DLAV at 48 hpf when compared to wild-type fish (Supplementary Figure S3A–C). Embryos showed ~50% pericardial edema at 72 hpf (*n* = 20 in wt and *n* = 19 in (*fli:fsta*) embryos) (Supplementary Figure S3D–F). The data are correlated with vasculature defects in overexpressed *fsta* embryos.

### 3.5. Specificity of fsta Effects

We showed that the overexpression of *fsta* driven by *fli1* promoter causes vascular defects. To test if any vascular phenotypes caused by loss of *fsta*, we knocked down *fsta* by using two different morpholinos, *fsta^ATG^* MO and *fsta^e3i3^* MO. We showed that a loss of *fsta* does not show obvious vascular defects (Figure 4C,C′ and Supplementary Figure S4A–C) from the injection of 2 kinds of morpholinos, suggesting the loss of *fsta* expression caused by morpholino inhibition specifically. In addition, there was no significant increase in ISV completion and the CVP loop formation in *fsta* morphants, suggesting that the loss of *fsta* is not sufficient for ISV and CVP growth (Figure 4C,C′). To examine as to whether the morpholino knockdown is *fsta*-sequence-specific, we tested the efficiency of *fsta^e3i3^* morpholino knockdown. By RT-PCR analysis, we showed that the injection of 8.0 ng of *fsta^e3i3^* morpholino diminished the normal fragments of *fsta* completely (Supplementary Figure S4E), while the injection of a lower dosage 1.0 ng *fsta* morpholino showed a decreased expression of *fsta*, suggesting the morpholino knockdown is *fsta*-specific. To further confirm the specificity of our morpholino experiments, we performed rescue experiments by the knockdown of *fsta* in *(fli:fsta)* embryos. The reduced expression of *fsta* indeed rescued ISV stalling by 30% in *(fli:fsta)* fish when compared with overexpressed *fsta* embryos (Figure 4B,D,E). In addition, the injection of *fsta* morpholino restored the honeycomb-like structure in *(fli:fsta)* fish to 80% at the CVP when compared to the overexpressed *fsta* embryos (Figure 4B′,D′,F).

### 3.6. Overexpression of fsta Remodeled the Expression of Vascular Markers

**Figure 5:**
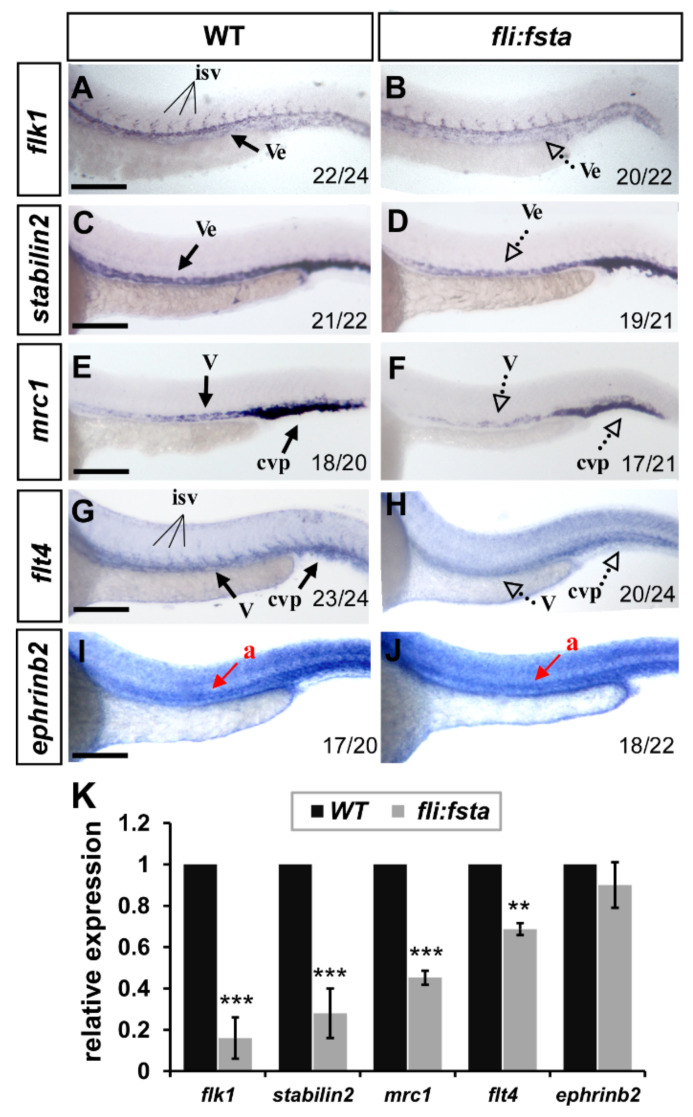
*fli,* driving *fsta*-overexpressing *(fli:fsta)* fish, remodeled the expression of vascular markers. (**A**–**J**) When compared to wild type controls (**A**,**C**,**E**,**G**,**I**), the expression of the pan-vascular markers *flk1* and *stabilin2* at the vessels (ve) and caudal vein plexus (cvp) were decreased in *(fli:fsta)*-overexpressed fish at 24 hpf (**A**–**D**). In addition, the expression of venous markers *mrc1* (**E**,**F**) and *flt4* (**G**,**H**) were reduced in the posterior cardinal vein (v) and caudal vein plexus (cvp) in *(fli:fsta)*-overexpressed fish at 24 hpf. The expression of the arterial marker *ephrinb2* (**I**,**J**) is not changed. Values on the bottom indicate the number of embryos exhibiting a phenotype per the total number of embryos analyzed. Scale bars represent 200 μm in (**A**–**J**). (**K**) Quantification of the relative expression level by qPCR assay showed a ~50 to 80% reduced expression in the vascular markers *flk1* (0.16 ± 0.1), *stabilin2* (0.28 ± 0.12), *mrc1* (0.45 ± 0.03), and *flt4* (0.69 ± 0.03) in *(fli:fsta)* embryos; the expression of *ephrinb2* (0.9 ± 0.11) in *(fli:fsta)* embryos is unchanged when compared to wild-type. qPCR data are presented as the mean ± S.D. *** Refers to *p* < 0.0001 and ** refers to *p* < 0.001 by an unpaired Student’s *t* test.

The growth defects and mis-patterns of ISV and CVP in *(fli:fsta)* fish suggest that *fsta* is important for vessel formation and likely regulates vessel identity. To test this idea, we examined the expression levels of several vascular markers, including *flk1*, *flt4*, *mrc1*, *stabilin2*, and *ephrinb2* by in situ hybridization and quantitative PCR. At 24 hpf, the expression of the pan-vascular markers *flk1* and *stabilin2* at the vessels and CVP were decreased in *(fli:fsta)*-overexpressing embryos; the expression of the venous/ISV markers *flt4* and *mrc1* was also reduced in overexpressed *fsta* fish when compared to the wild type controls. However, the expression level of the arterial marker *ephrinb2* had not changed (Figure 5). The decreased level of the vascular marker expression was quantified by qPCR and was consistent with the changed expression in *(fli:fsta)* embryos (Figure 5K). These results suggest that the overexpression of *fsta* disrupts the expression of several vascular genes and impairs vessel formation.

### 3.7. Knockdown of fsta Rescues the Vascular Defects in isl2 or nr2f1b Morphants

**Figure 6:**
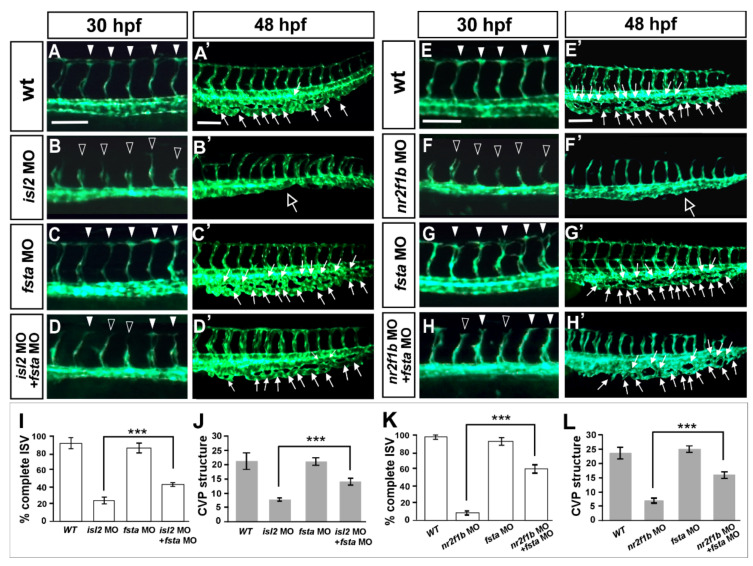
Knockdown of *fsta* rescues the vascular defects in *isl2* and *nr2f1b* morphants. When compared to vascular patterns in wild type embryos (**A**,**A′**,**E**,**E′**,), the loss of *fsta* by morpholino injection does not cause obvious vascular defects (**C**,**C’**,**G**,**G’**), and the knockdown of *isl2* or *nr2f1b* showed ISV growth defects at 30 hpf (**B**,**F**) and CVP mis-patterning (hollow arrows in (**B′**,**F′**)). Double knockdown of *fsta* and *isl2* (*fsta* MO *+ isl2* MO) or *fsta* and *nr2f1b* (*fsta* MO *+ nr2f1b* MO) showed less ISV stalling (**D**,**H**) when compared to *isl2* MO or *nr2f1b* MO. (**I**–**L**) Quantitative results showed the significant rescue effect of *isl2* MO or *nr2f1b* MO by *fsta* morpholino injection. Quantitated results for *isl2* MO rescue experiments in wt (*n* = 10), *fsta* MO (*n* = 18), *isl2* MO (*n* = 13), and *fsta* MO *+ isl2* MO (*n* = 15). For the quantification of *nr2f1b* MO rescue experiment: wt (*n* = 10), *fsta* MO (*n* = 18), *nr2f1b* MO (*n* = 15), *fsta* MO *+ nr2f1b* MO (*n* = 13). These data are consistent with our microarray results; *isl2* and *nr2f1b* negatively regulate *fsta* expression to control ISV/vein development. Data are represented as means ± S.E. *** refers to *p* < 0.0001 by an unpaired Student’s *t*-test. Scale bars represent 100 μm in (**A**–**H**,**A′**–**H′**).

Overexpressing *fsta* gene phenocopy shows the loss of *isl2/nr2f1b,* which suggests that *fsta* is negatively regulated by *isl2/nr2f1b*. To confirm the genetic relation between *fsta* and *isl2/nr2f1b*, we hypothesized that if there was a reduction in the expression level of *fsta* in *isl2* or *nr2f1b* morphants, we should see the rescue effects. Figure 6 shows that the loss of *fsta* does not cause obvious vascular defects (Figure 6C,C′), and the knockdown of *isl2* shows growth defects in the ISV and CVP (Figure 6B,B′). The double knockdown of *fsta* and *isl2* shows less ISV stalling and restored the CVP structure (Figure 6D,D′) when compared to the *isl2* MO. Quantitative results showed the significant rescue effect of *isl2* MO by *fsta* morpholino injection (Figure 6I,J). Similar rescue results and quantitative data are shown to restore vascular defects in *nr2f1b* morphants (Figure 6E–H,E′–H′,K,L). The data consistent were with our microarray results; *isl2/nr2f1b* negatively regulates *fsta* expression to control ISV/CVP development.

### 3.8. Follistatin a Regulates BMP Signals to Control Vascular Patterning

**Figure 7:**
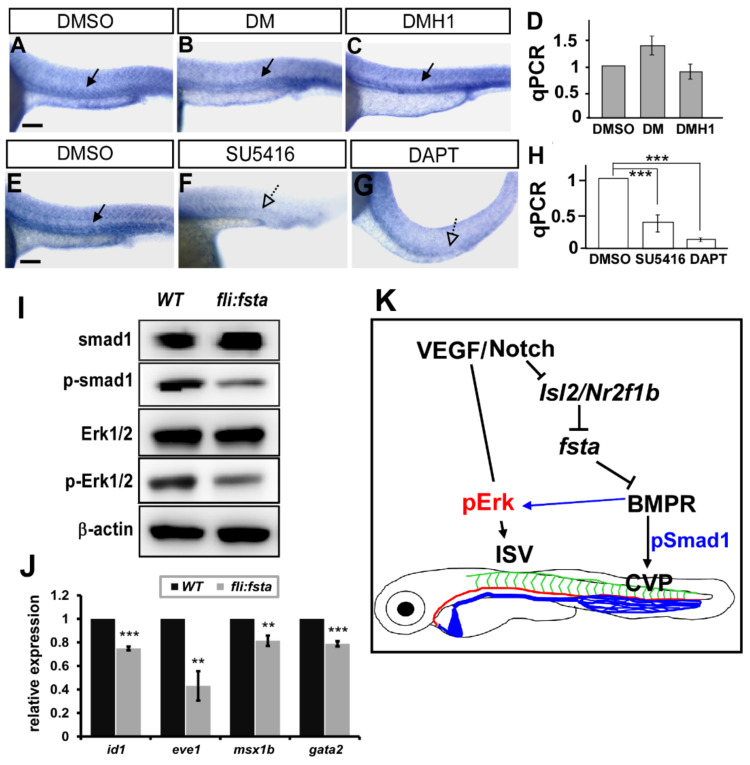
*Fsta* regulates BMP signals to control vascular patterning. (**A**–**D**) Inhibition of VEGF or Notch signals by SU5416 or DAPT treatment reduced the expression of *fsta* compared to DMSO-treated control. (**E**–**H**) Inhibition of BMP signals by DM or DMH1 treatment did not reduce the expression of *fsta* in embryos by in-situ hybridization and qPCR analysis. (**I**) Representative western blot assays showed the reduced phosphorylation of smad1 and ERK1/2 in *(fli:fsta*) embryos. β-actin serves as loading controls. (**J**) The decreased expression of BMP regulated genes *id1*, *eve1*, *msx1b* and *gata2*. qPCR data are presented as the mean ± S.D. *** refers to *p* < 0.0001 and ** refers to *p* < 0.001 by an unpaired Student’s *t*-test. Scale bar represents 200 μm in (**A**–**C**,**E**–**G**). (**K**) Schematic drawing shows the genetic regulation of *fsta*, *isl2/nr2f1b*, and multiply signals. Our data suggest that *fsta* functions in ISV growth and CVP patterning mediated by BMP signal pathways.

Vascular development in zebrafish is mainly mediated by VEGF/Notch and BMP signals. To reveal the genetic interaction between *fsta* and multiple signals, we analyzed the expression of *fsta* after the administration of chemicals. We inactivated Notch and VEGFR2 signaling by DAPT and SU5416 treatment, respectively, and showed that the expression of *fsta* is downregulated dramatically at the vessels and CVP (Figure 7E–H). The data suggest that a genetic interaction between *fsta* and VEGFR2–Notch signals exists. Fsta is a BMP antagonist related to axis formation during development, and BMP signaling has been shown to be involved in venous angiogenesis. To test whether Fsta regulates BMP signals to control vascular patterning, we used the small molecules DM or DMH1 to inhibit BMP signals. We showed that inhibition of BMP signals by DM or DMH1 treatments impaired the CVP structures similar to the *(fli:fsta)*-overexpressed embryos (data not shown). However, DM or DMH1 treatment did not reduce the expression of *fsta* in embryos (Figure 7A–D), suggesting it is upstream of the BMP signals. To test if overexpression of *fsta* disrupts BMP signals, we performed Western blot analysis to examine the signal molecules in the pathways. We showed the reduced phosphorylation level of SMAD1 and Erk1/2 in *(fli:fsta)* embryos (Figure 7I). Since BMP receptors also regulate ERK signals, our data suggest that *fsta* regulates CVP patterning via BMP–SMAD pathways and contributes to ISV growth mediated by BMP–ERK pathways (Figure 7J).

## 4. Discussion

The genetic networks that control vascular patterning during the development are not fully understood. We previously identified the transcription factors Isl2 and Nr2f1b, which are required for the specification of the vein and ISV growth, mediated by the Notch pathway in zebrafish. In this study, our microarray analysis suggested that *nr2f1b* and *isl2* cooperatively regulate vascular development. We further analyzed the functional relevance of one potential target from array hits, *fsta*. We showed that *fsta* is highly conserved among vertebrates and its expression in developing vessels suggests its roles in the vasculature. We showed that *fsta* functions in vascular development downstream of *isl2/nr2f1b*, because of the following: a) overexpressing *fsta* in zebrafish embryos causes vascular defects in the growth of ISV and CVP, which mimics the phenotype of *isl2/nr2f1b* morphants; and b) the knockdown of *fsta* can partially rescue the vascular defects in *isl2* or *nr2f1b* morphants. In addition, we showed that the vascular defects in *(fli:fsta)* embryos remodel the changed expression of vascular markers. Finally, our data indicated that Fsta–BMP signals control vascular patterning, mediated by the phosphorylation of SMAD and Erk. We conclude that Fsta–BMP signaling plays an important role in the regulation of vascular patterning, which interacts with *isl2/nr2f1b* and multiple signals. Our study also provides useful information for regulating networks in vascular development.

In this study, we used a genome-wide microarray analysis to identify potential gene functions in vascular development acting downstream of Isl2/Nr2f1b. We further characterized the role of *fsta* in the vasculature that has not been reported before, although many studies have shown that follistatin functions in skeletal muscle development and growth and even shows potential medical applications for muscle disorders. Our study demonstrated that *isl2/nr2f1b* regulates the growth of ISV and CVP formations, which are likely medicated by Fsta–BMP signals. Therefore, it may be possible to use *fsta* as a therapeutic agent in vascular-related diseases. Although our microarray suggests the expression changes of many genes in response to *nr2f1b* and/or isl2, whether they are regulated directly or indirectly by Isl2/Nr2f1b will be interesting to address. Promoter analysis of potential targets that contain Isl2 or Nr2f1b binding sites as well as luciferase analysis or chromatin immunoprecipitation (ChIP) are potential methods to examine whether those targets are controlled by Isl2/Nr2f1b directly.

Transcriptome analysis in zebrafish embryos has been shown as a powerful tool to identify many novel genes important for vascular development [53,54,55,56]. Our microarray strategies successfully allowed us to identify a genes’ potential function in the process of artery–vein specification and tip–stalk cell specification downstream of Isl2/Nr2f1b. In situ hybridization confirmed the expression pattern of several potential targets *fsta*, *mmp2*, *foxo3b*, *lnx1*, *ftr82*, *cpn1*, etc., in the vessels and suggests their role in the vasculature. Thus, many of the genes are novel and can be served as novel vascular markers and/or therapeutic targets. In addition to the characterization of *fsta* in this study, the *Gtpbp1l* (*GTP-binding protein 1-like*) gene encodes a putative GTPase and belongs to the GTP-binding protein superfamily. The deduced amino acid sequence exhibited the highest overall homology with human GTPBP1 [57]. GTPases are key proteins in many critical biological processes, including hormonal and sensory signals, ribosomal protein synthesis, cytoskeletal organization, and signal transduction cascades and motility [58]. Small GTPase Rap1 has been shown to promote VEGFR2 activation and angiogenesis [59]. Rho guanine exchange factors have reported functions in angiogenesis and the vasculature [60]. However, GTPBP groups are not known to function in vessels.

Mmp2 is a known metalloprotease involved in extracellular matrix integrity, which is important for angiogenesis and cell migration. In addition, Mmp2 acts to degrade collagen type IV and permits cell migration through tissues [61,62]. Interestingly, it has been shown that Mmp2 controls branching morphogenesis, mediated by FGF signaling [63], which extends our understanding of pathway networks in angiogenesis. Foxo3b (forkhead box O3b transcription factor) has been shown to function in angiogenesis and postnatal neovascularization by the inhibition of eNOs [64]. Cpn1-encoded carboxypeptidase N1 has been shown important for zebrafish liver development [65]. In addition, Davis et. al. showed carboxypeptidase N as an enzyme that is responsible for the C-terminal cleavage of SDF-1a (stromal cell-derived factor-1alpha) in circulation, which is important for hematopoiesis, lymphocyte homing, pre-B-cell growth, and angiogenesis [66]. Lnx1 is named as a ligand of numb-protein X1, and has been reported as an E3 ubiquitin ligase. The disruption of tfigurhe ligand of numb protein X expression can promote a TGF-β-induced epithelial-to-mesenchymal transition of epithelial cells [67], but no vascular functions have been reported yet. It is noteworthy that some interesting targets or their related family members shown in this list have recently been identified as being functional in the vascular development, such as *mmp2*, *aqu12*, *foxo3b*, *cdkn1a*, *pik3r1*, *hdac4*, *igf2*, *halpn1b*, etc., [68,69], suggesting that those target genes regulated by Isl2 and/or Nr2f1b are promising target genes that are important for vascular development.

Thus, our data provide useful information for the networks that regulate vascular development. Our data suggest that many novel genes, such as *fsta*, *lnx1*, *ftr82*, *itm2bb*, *gtpbpl1*, *cpn1*, *stap2b*, etc., have potential vascular functions that have not been previously described. In addition, many interesting signal pathways likely involved in those process have not been fully documented, such as PI3K/Akt signals, BMP signals, Wnt signals, and GTPase signals. Thus, our study expands the vascular fields of ISV and CVP patterning, as well as vein and tip identity.

## 5. Conclusions

We aimed to identify the potential downstream targets of Islet2 and Nr2f1b transcription factors, which are essential for vascular development. By employing microarray strategies, genetic manipulation, and biochemical assays in transgenic zebrafish, we have characterized the novel role of the *follistatin a (fsta*) gene for vessel formation downstream of *isl2*/*nr2f1b*. Besides, we revealed that Isl2/Nr2f1b regulate ISV growth and CVP formation medicated by Fsta–BMP signaling. To our knowledge, the novel function of *fsta* in the genetic network during vascular development has never been explored. Understanding the molecular mechanisms underlying the *isl2/nr2f1b*–mediated control of vascular development will also assist in the discovery of novel biomarkers and/or therapeutic targets for vascular diseases.

## Data Availability

Not applicable.

## Supplementary Materials

The following supporting information be downloaded at: https://www.mdpi.com/article/10.3390/biomedicines10061261/s1, Table S1: Primer sequences for qPCR experiments; Table S2: Primer sequences used to generate in-situ hybridization probes; Figure S1: Sequence comparison and analysis of Fsta protein; Figure S2: Vascular defects in *fsta* overexpressed embryo is not due to apoptosis; Figure S3: Overexpression of *fsta* results in pericardial edema and circulation defect; Figure S4: Morpholino-knockdown efficiency of *fsta* in zebrafish embryos.
