# Supplementary material for: Identification of Novel Vascular Genes Downstream of Islet2 and Nr2f1b Transcription Factors
Source: Biomedicines. 2022 May 27;10(6):1261. doi: 10.3390/biomedicines10061261 (PMC9220758; doi:10.3390/biomedicines10061261)
Supplement: Supplementary file 1 [file biomedicines-10-01261-s001.zip › biomedicines-1651007-supplementary.pdf]

biomedicines

Supplementary Materials

Identification of novel vascular genes downstream of Islet2 and Nr2f1b transcription factors

Ru-Fang Li [1,†], Yi-Shan Wang [1,2,3,†], Fu-I Lu [4,5], Yi-Shan Huang [1,2], Chien-Chih Chiu [1,6], Ming-Hong Tai [1,7] and Chang-Yi Wu [1,2,3,5,8,*]

Supplementary Methods:

Morpholino efficiency

The efficiency of morpholinos that cause mis-splicing was determined using reverse-transcriptase polymerase chain reaction (RT-PCR) with primers spanning either side of the targeted exon. RNAs extracted from both injected and uninjected control embryos and reverse transcript to cDNA. For *fsta* MO efficiency and specificity test, primers. *fsta*-mo-f：5′-GGACTGTGGTCCTGGAAAGA-3′ and *fsta*-mo-r：5′ -GATGGTTCTACCGAGCAAGA-3′ were used, while for GAPDH (a loading control), primers GAPDH_F 5′- TGCTGTAACCGAACTCATTGTC-3′ and GAPDH_R 5′- CAAGCTTACTGGTATGGCCTTC-3′ were used. The results were visualized by running 5 µl of the PCR reaction on 1 % agarose gel.

Apoptosis assays

Acridine orange staining (AO staining): Dechorionated embryos were soaked in E3 medium containing 2 µg/ml acridine orange for 30 minutes. After washing with fresh E3 medium six times, embryos were mounted in 3% methylcellulose with 5% tricaine and photographed.

TUNEL assay: TUNEL assay was performed using in situ cell death detection kit (Roche) according to manufacturer's instructions. Briefly, embryos were fixed in 4% paraformaldehyde (PFA) and stored at -20°C in methanol. After rehydration, permeabilization with proteinase K (Roche), embryos were re-fixed in 4% PFA for 15 minutes, then treated with 3% H_2O_2 in 0.1% PBT to eliminate endogenous peroxidase, following by incubation with 45µl of TUNEL (TdT-mediated dUTP-X nick end labeling) label plus 5µl of TUNEL enzyme solution for 3 hours at 37°C in the dark. Embryos were then washed with PBT, blocked with 5% Normal Sheep Serum (NSS) in PBT, incubated with peroxidase (POD) conjugated anti-fluorescein antibody (Roche) over night at 4°C in the dark, washed four times in PBT, and visualized using DAB substrate

Table S1: Primer sequences for qPCR experiments

Gene	Primer sequences
fsta	fsta-qf:5'-CCCAGAGGTCATGTCTCCAG-3'<br>fsta-qr:5'-CCGGTATGTTTTTCCATCTGA-3'
ephrinb2	ephrinb2-qf:5'-CTGGAACACCACGAACACC-3'<br>ephrinb2-qr:5'-CACACGTGGGCAAACTATGT-3'
mrc1	mrc1-qf:5'-CTAGCAAGCCTGAAGGTGCC-3'<br>mrc1-qr:5'-TGAGAGGCTGGGTAGTTGGG-3'
flt4	flt4-qf:5'-ACTCGGGTTATTACCGCTGCTTCT-3'<br>flt4-qr:5'-TGGATGCTCTGGGTCTCGAACAAA-3'
flk1	flk-qf:5'-ACTTTGAGTGGGAGTTTCATAAGGA-3'<br>flk-qr:5'-TTGGACCGGTGTGGTGCTA-3'
stabilin2	stabilin-qf:5'-GGGCTTCCAATACCAACTGG-3'<br>stabilin-qr:5'-CCTGGTTGCACAGACAGACC-3'
id1	id1-qF: 5'-CGCGGATCCGAGAGCATGTGGTGAACTGT-3'<br>id1-qR: 5'-CCGGAATTCAGGTAGGTCCCACAACTTTC-3'
eve1	eve1-qF: 5'-GGGTAGTCTCTCTGGGTTTT-3'<br>eve1-qR: 5'-GAATAGAGAGCTGGTTGTGG-3'
msx1b	msx1b-qf: 5'-CCAGCAGGTCGCGTGTTCTCC-3'<br>msx1b-qr: 5'-GCTTGCGTAAGGTGCACGGC-3'
gata2	gata2-qF: 5'-GCTGAATGTGTGAACTGTGGA-3'<br>gata2-qR: 5'-TGGCTTGATAAGGGGTCTGT-3'
ef1a	ef1a-2f:5'-TCAACGCTCAGGTCATCATC-3'<br>ef1a-2r:5'-GATGTGAGCAGTGTGGCAATC-3'
β-actin	β-actin_qf:5'-CTCTTCCAGCCTTCCTTCCT-3'<br>β-actin_qr:5'-CTTCTGCATACGGTCAGCAA-3'

Table S2: Primer sequences used to generate in-situ hybridization probes

in-situ probe primers	Sequence
mmp2_ISH_f	5'-AGCATGGGTCTTCCTTCAGA-3'
mmp2_ISH_rT7	5'-TGTAATACGACTCACTATAGGGCCATCATGCAACAAACCCTA-3'
foxo3b_ISH_f	5'-CTGACCTGACTGGCACAATG-3'
foxo3b_ISH_rT7	5'-TGTAATACGACTCACTATAGGGTCTTCCACTGGGCAGTCTTT-3'
cpn1_f:	5'-TCAGAAAGCTGGCAAAGACA-3'
cpn1_rT7	5'-TAATACGACTCACTATAGTGAAAAACCCGCTTTTATGC-3'
ftr82_f	5'-TGCAGCCGTACTCTGACAAC-3'
ftr82_rT7	5'-TAATACGACTCACTATAGTGTCCATCAATCATGCCTTC-3'
itm2bb_f:	5'-AGAAGCTGGACGACACCAGT-3'
itm2bb_rT7	5'-TAATACGACTCACTATAGTGACGATGCTTCTTCACTCTG-5'
gtpbp1l_f:	5'-GCAGTCCAGCAAGAAACCTC-3'
gtpbp1l_rT7	5'-TAATACGACTCACTATAGCGGTTTTATCCACCGTGATT-3'
scarb2-f1	5'-GGCCTTCTCAAACTGCCATA-3'
scarb2-r1T7	5'-GGGGTAATACGACTCACTATAGGGCCGACATGCTTGCTGAAATA-3'
lnx1_f	5'-GGTCGGATCACTCTCACCAT-3'
lnx1_rT7	5'-TAATACGACTCACTATAGGGAGGATACCCGTCTCATGTGC-3'
cx32.2-f1	5'-TGTAGGAGGATCAGTCGGAGA-3'
cx32.2-r1T7	5'-GGGGTAATACGACTCACTATAGGGTTTATTTGTGTCCCCCTAGCA-3'
sfrp1a-f1	5'-TCCCTTGCATCTTTGTCCCT-3'
sfrp1a-rT7	5'-TAATACGACTCATATAGGCGCACATGTGATCCAGGAT-3'
WASF1-f1	5'-ACCAACATCTCTCTGGCCAA-3'
WASF1-rT7	5'-TAATACGACTCATATAGAGTGTCCTGCAGCATCTTCT-3'
nsdhl-f1	5'-TGATGGCGCTCTCTTTCAGA-3'
nsdhl-rT7	5'-TAATACGACTCATATAGTCCCATAGACCAGCGCATAG-3'
stap2b-f1	5'-TTTCACTGTGGAACGAGGAG-3'
stap2b-r1T7	5'-GGGGTAATACGACTCACTATAGGGCAAAGTTGAACAGGAACAGACAA-3'

T7 RNA polymerase binding sequences are labeled underline.

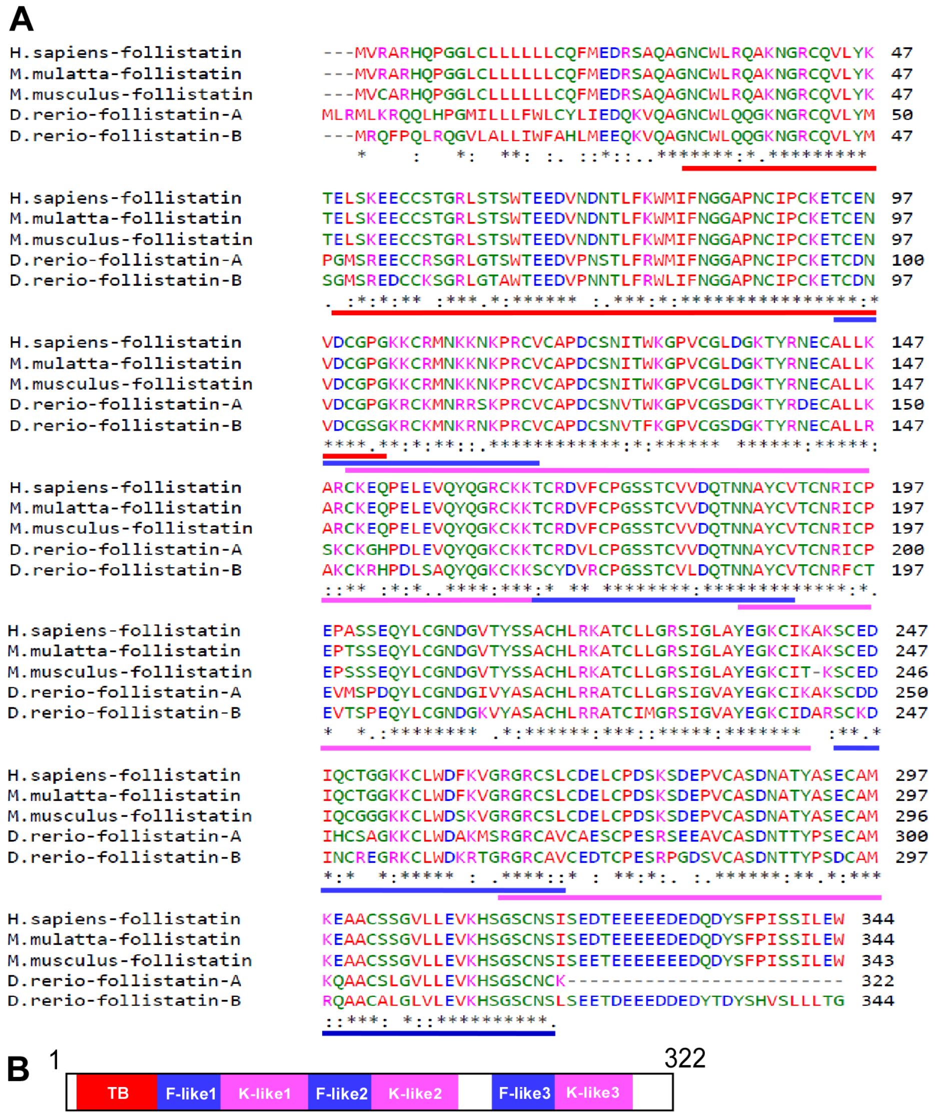

C

H.s_fst	100				
M.mul_fst	99	100			
M.mus_fst	97	97	100		
D.r_fsta	75	75	75	100	
D.r_fstb	66	67	67	79	100
	H.s_fst	*M.mul_fst*	*M.mus_fst*	*D.r_fsta*	*D.r_fstb*

Figure S1. Sequence comparison and analysis of Fsta protein.
(A). Sequences alignment of follistatin amino acids among vertebrates, human (*Homo sapiens* NP_037541.1-*fsta*), monkey (*Macaca mulatta* XP_001095829.2-*fsta*), mouse (*Mus musculus* NP_032072.1-*fsta*) and zebrafish (*Danio rerio* NP_571112.3-*fsta* and NP_001034720.1-*fstb*) by using ClustalW2 (http://www.ebi.ac.uk/Tools/msa/clustalw2/). (*) represents identical amino acids and (. or :) represents amino acid similarity. **(B)** Schematic drawing of zebrafish follistatin a contains a putative TB ligand binding domain (similarity = 95%), three F-like domains and three Kazal-like domains (similarity = 98%). **(C)** Conservation percentage of Fsta amino acid identity matrix among vertebrates and zebrafish.

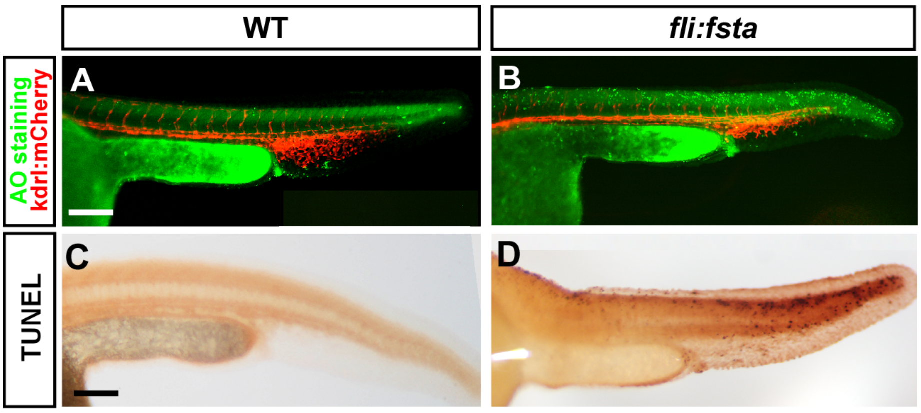

Figure S2. Vascular defects in *fsta* overexpressed embryo is not due to apoptosis.
(A-D) AO staining in *Tg(kdrl:mCherry)* fish and TUNEL assay were used to detect apoptotic cells in wild type **(A, C)** and overexpressing *fsta* embryos **(B, D)**. Increased apoptotic cells were observed on the skin and at the epidermis of dorsal tail areas, but not in the region of vasculartrure (red fluorescence) in *(fli:fsta)* embryos compared to wild type controls. The scale bars are 200 μm.

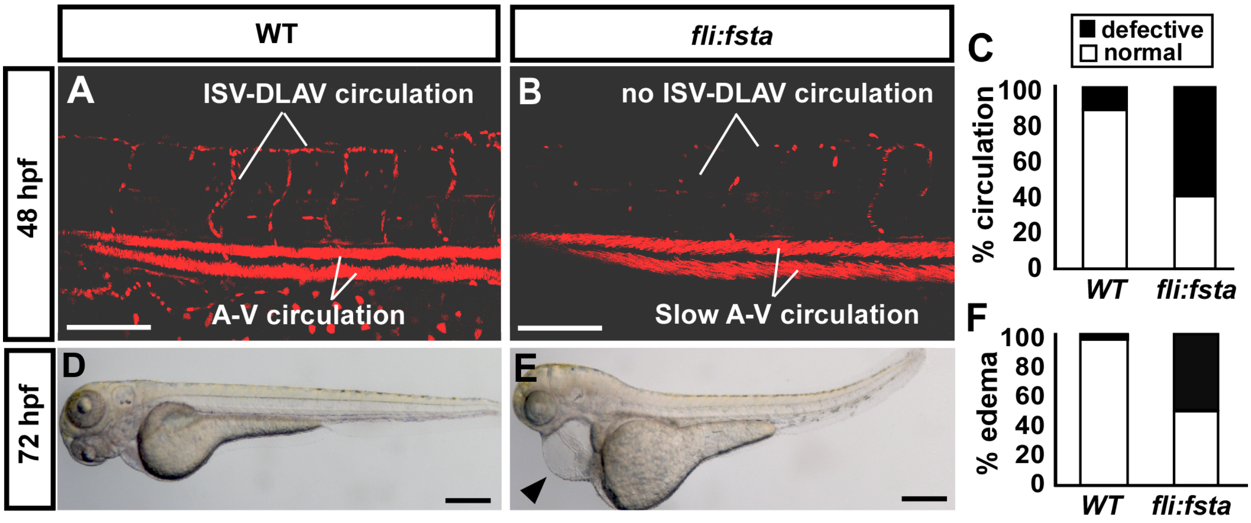

Figure S3. Overexpression of *fsta* results in pericardial edema and circulation defect.
(A-C) Overexpression of *fsta* in transgenic *Tg (gata1:dsRed)*^{sd2} embryos with dsRed-labeled blood cells allow us to observe circulation. Overexpression of *fsta* embryos showed 60% circulation defects with slower blood flow in artery/vein and/or less-to-no circulation at ISV/DLAV at 48 hpf compared to wild-type fish **(A, B)**. **(C)** Circulation defects at the ISV-DLAV and/or aorta-vein in the trunk region are quantitated in wt (n=20) and (*fli:fsta*) embryos (n=20) at 48 hpf. **(D-F)** Representative edema fish and quantitative results from three independent experiments showed ~50 % of (*fli:fsta*) embryos (n=19) with mild to severe pericardial edema compared to wt (n=20). The scale bar in A-B represent 100 µm and in D-E is 500 µm.

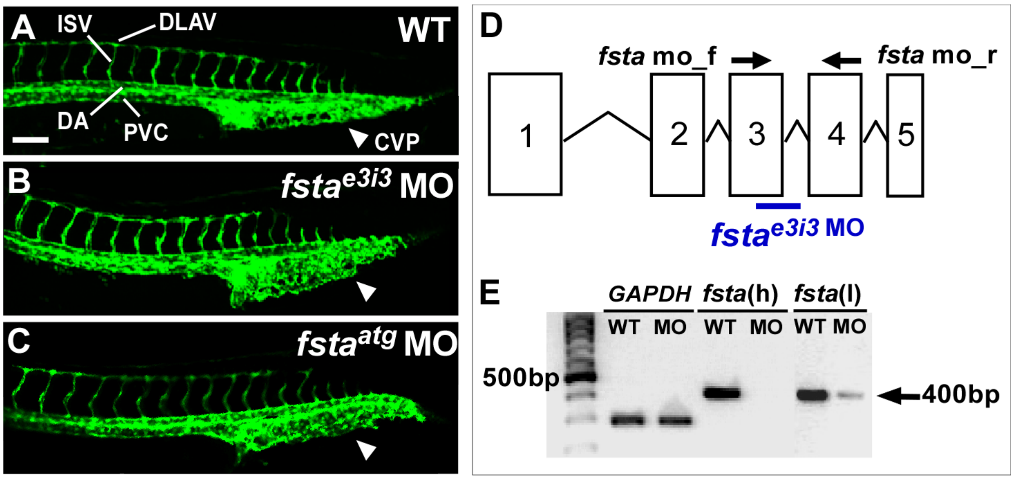

Figure S4. Morpholino-knockdown efficiency of *fsta* in zebrafish embryos.
(A-C) Compared to wild type embryos, knockdown of *fsta* by either splicing morpholino (*fst*^{e3i3} MO) **(B)** or translational block morpholino (*fsta*^{atg} MO) **(C)** injection does not cause obvious vascular defects in ISV and CVP at 30hpf. The scale bar in A-C represent 100 μm (**D**) Scheme shows *fsta* morpholino targeting the pre-mRNA structure of *fsta* and suggested mis-splicing fragment which can be detected by a *fsta*_mo_f and *fsta*_mo_r primer set. **(E)** cDNA from uninjected controls or *fsta* morphants (injected with high dosage 8.0 ng (h) or low dosage 1.0 ng (l) morpholino) underwent PCR with primers for the housekeeping control gene (*GAPDH*), or for the *fsta* gene. *GAPDH* levels in *fsta*^{e3i3} morphants are unchanged (245bp) while the amount of wild-type *fsta* product (400bp) showed dose-dependent decrease.
